# Supplementary material for: Complexome profiling of the Chlamydomonas psb28 mutant reveals TEF5 as an early PSII assembly factor
Source: Plant Cell. 2025 Mar 17;37(6):koaf055. doi: 10.1093/plcell/koaf055 (PMC12164590; doi:10.1093/plcell/koaf055)
Supplement: koaf055_Supplementary_Data [file koaf055_supplementary_data.zip › psb28_interactive.zip!/plots/intensity/qualityPlots/peptides/ATPVL1_Cre06.g257450.t1.2.html]

### Cre06.g257450.t1.2

Trivial name: ATPVL1  
SeqCoverage [median]: 17.00  
Mass: 17.59 kDa  
  
 

| Sequence | MissedCleavage | Mass [Da] | wtIntensity | muIntensity | Score |
| --- | --- | --- | --- | --- | --- |
| AGASAAAPPAAL | 0 | 966.51 | 841.62 | 1482.02 | 122.74 |
| SGVGIASMGVMRPELVMK | 1 | 1860.96 | 42253.90 | 110472.64 | 112.67 |
|  |  |  | 43095.5 | 111954.7 |  |
